# Mindfulness as Predictor of Itch Catastrophizing in Patients With Atopic Dermatitis: Results of a Cross-Sectional Questionnaire Study

**DOI:** 10.3389/fmed.2021.627611

**Published:** 2021-03-26

**Authors:** Kjell Lüßmann, Kerry Montgomery, Andrew Thompson, Uwe Gieler, Christoph Zick, Joerg Kupfer, Christina Schut

**Affiliations:** ^1^Institute of Medical Psychology, Justus-Liebig-University, Giessen, Germany; ^2^Department of Psychology, University of Sheffield, Sheffield, United Kingdom; ^3^South Wales Doctorate in Clinical Psychology, Cardiff University, Wales, United Kingdom; ^4^Uwe Gieler, Department of Dermatology, Justus-Liebig-University, Giessen, Germany, Clinic of Psychosomatics, Vitos Clinic, Giessen, Germany; ^5^Department of Dermatology, Rehabilitation Center Borkum Riff, Borkum, Germany

**Keywords:** itch, atopic dermatitis, mindfulness, rehabilitation, itch catastrophizing

## Abstract

Itch and worry about itch are predominant symptoms in atopic dermatitis (AD). Mindfulness refers to paying attention in a certain way, non-judgementally and on purpose. In patients with chronic pain, which shares several similarities with chronic itch, a significant relationship between pain intensity, mindfulness and pain catastrophizing has been found. The aim of this study was to investigate whether itch intensity and mindfulness are related to itch catastrophizing in AD patients. Participants receiving treatment for AD (*n* = 155; 58 male; mean age: 46.5 ± 12 years) completed measures of itch-related catastrophizing (Itch Cognitions Questionnaire; ICQ) and mindfulness (Comprehensive Inventory of Mindfulness; CHIME) during their stay at a rehabilitation center in Borkum, Germany. In addition to other variables, their average itch intensity during the last 2 weeks was assessed by means of a visual analog scale. A positive relationship between itch intensity and itch catastrophizing was found (*r* = 0.409; *p* < 0.01). Moreover, the mindfulness scales “acting with awareness,” “accepting and non-judgemental orientation,” and “non-reactive orientation” were negatively related to itch catastrophizing. A linear regression analysis revealed that itch intensity in combination with “acting with awareness” was able to explain more than 27 % (corrected *R*^2^ = 0.274; *p* < 0.001) of the variance of itch catastrophizing. Thus, itch intensity and certain facets of mindfulness were associated with itch catastrophizing in AD patients. Psychological interventions aiming to increase acting with awareness might have a buffering effect on itch catastrophizing, which in turn could lead to lower itch intensity in patients with AD. Future RCTs should test this hypothesis.

## Introduction

Itch or pruritus is an unpleasant bodily sensation usually accompanied by scratching behavior (1). When itch lasts longer than 6 weeks, it is regarded as chronic (2). Chronic itch is a common symptom in patients with skin diseases like atopic dermatitis (AD) (3). It has been shown that itch and scratching behavior play a role in the maintenance and exacerbation of AD as the mechanic stimulation of the skin through scratching can provoke increased inflammation whilst also removing any topical treatments that have been applied (4). Further inflammation then subsequently triggers an additional itch sensation.

In addition to the direct behavior of scratching, psychological, and social factors play an important role in the maintenance and worsening of the itch-scratch cycle in AD. The biopsychosocial model of chronic itch (5) proposes that internal (e.g., personality) and external factors (e.g., stress) can lead to illness cognitions, social reactions, and behaviors, which can increase physiological responses (e.g., activation of certain brain areas) leading to itch (5). Studies have reported that excessive worrying about the skin condition is associated with the severity of physical symptoms in AD and psoriasis (6). Schut et al. (7) found a significant correlation between perceived stress, cognitive coping, and itch intensity in patients with AD. In this study, the relationship between stress and itch was mediated by itch-related cognitions and patients, who reacted to stress with negative itch-related cognitions reported higher itch intensities than patients with the opposite cognitive reaction (7).

Mindfulness has been defined as “paying attention to the present moment, non-judgmentally and on purpose” [(8), p4]. Sometimes, the term mindfulness is understood as synonym of meditation. However, psychological models of “mindfulness” posit that the construct is made up of a number of different facets (9). Further, these facets are believed to be present to varying degrees in all individuals and as such are thought to some extent to represent “personality” variables. Thus, mindfulness comprises more than just emotional awareness, but also e.g., awareness of surrounding others, things, acting consciously (9). These facets of mindfulness have been found to be significantly associated with wellbeing (10). As postulated by Shenefelt (11) mindfulness could help via psychoneuroimmunological pathways to reduce itch. In a dermatology sample mindfulness explained a significant proportion of the variance in social anxiety, anxiety, depression, and health-related quality of life after controlling for subjective severity of the skin condition (12). Moreover, there is some evidence that mindfulness based interventions can have a positive effect on the skin status and wellbeing of patients with psoriasis that can also be accompanied by itch [e.g., (13–15)]. Whether also participation in a *short* 2-week-mindfulness-based intervention delivered at a German rehabilitation clinic is beneficial in patients with the chronic, in many cases itchy skin disease psoriasis is investigated at the moment (16).

Itch shares some similarity with pain (17) as both are unpleasant sensations with common pathophysiological features (18). In patients with chronic pain, mindfulness predicted pain catastrophizing and moderated the relationship between pain intensity and pain catastrophizing (19). Increasing levels of mindfulness can be effective in reducing the experience of pain (20), which may have important implications for people living with AD given the significant association between itch and chronic pain (21). Therefore, the current study aims to investigate the relationship between itch intensity, mindfulness and itch catastrophizing in patients with chronic itch. It is hypothesized that in addition to itch intensity, levels of mindfulness will be significant predictors of itch catastrophizing. In this context, catastrophizing refers to a negative way of thinking about the bodily symptom itch, e.g., the course or consequences of it. Such negative thoughts measured in this study are “The itching will never stop,” “I will scratch myself again until I look horrible” (22).

## Materials and Methods

### Inclusion and Exclusion Criteria and Procedure

Participants were receiving treatment at the rehabilitation clinic on Borkum, Germany and were assessed for eligibility and recruited to the study by KL (medical student) and CZ (Consultant and Lead Dermatologist). Inclusion criteria included being between the ages of 18–70 and having a verified diagnosis of atopic dermatitis of at least 1 year duration. In order to ensure that participants could complete the survey, proficiency in the German language was also required. Exclusion criteria included having a comorbid severe psychological or psychiatric condition or another skin disease associated with itch, or having another long-term disease which was in the focus at the time of investigation. Participants were asked to complete the questionnaires and return them during their 1st week at the clinic.

### Ethics

Ethical approval was gained at the University of Gießen (AZ: 210/15). Information on the study was provided to all participants who signed the consent form before filling in the questionnaires.

### Measures

#### Mindfulness

The Comprehensive inventory of mindfulness experiences (CHIME) (9) was used to measure different facets of mindfulness. It provides a multi-dimensional assessment of mindfulness and consists of 37 items measured on a six-point scale (1-6). Participants were asked to rate levels of mindfulness over the last 2 weeks. Eight subscales of mindfulness were measured; (1) awareness toward internal experiences, (2) awareness toward external experiences, (3) acting with awareness, (4) accepting and non-judgemental orientation, (5) non-reactivity, (6) openness to experiences, (7) relativity of thoughts, and (8) insightful understanding.

#### Itch Catastrophizing

The Itch Cognitions Questionnaire (22, 23) was used to measure itch catastrophizing. Participants were asked to rate the frequency of thoughts they had regarding itch over the past 2 weeks, for example “*The itching will get worse and worse*.” The questionnaire consists of 20 statements measured on a five-point Likert scale ranging from 0 (thought never occurs) to 4 (the thought usually occurs) which can be combined to the two scales “itch catastrophizing” and “itch coping.” In the current study we were only interested in the scale “itch catastrophizing.”

#### Itch Intensity

Itch intensity during the last 2 weeks was measured by a visual analog scale ranging from 0 to 10. The item to measure itch was “Please evaluate on this scale how intense your itch was on average during the last 2 weeks.”

#### Atopic Dermatitis Severity—Self Report

The Patient Orientated SCORing Dermatitis Index (PO-SCORAD) (24) was used to measure the self-rated severity of AD. Participants indicate the area of the body affected (POSCORAD-A), the severity of the condition including redness, scratch lesions, skin dryness, edema, and skin thickening (POSCORAD-B). In addition, using visual analog scales participants indicated the severity of itching and insomnia (POSCORAD-C). As for the SCORAD, also POSCORAD scores can range from 0 to 103 with higher scores indicating a more severe AD. SCORAD-scores below 25 indicate a mild AD, scores between 25 and 50 indicate a moderate AD and scores > 50 indicate a severe AD (25). In former studies, it was shown that SCORAD scores rated by clinicians and PO-SCORAD scores rated by patients highly correlate (24, 26).

### Other Variables

Besides the before mentioned variables, further data on alexithymia, self-rated causes of itch, sociodemographic variables and comorbidities were gathered which are included in the doctoral thesis of the first author of this article.

### Statistical Analysis

Statistical analyses were conducted using SPSS 24. Due to the fact that variables were not normally distributed in all cases, Spearman-Rank-correlations were conducted in addition to Pearson correlations to investigate the associations between mindfulness, itch catastrophizing, and itch intensity. These analyses did not reveal substantially different results (differences in *r*'s < 0.045). After testing whether the assumptions for a linear regression analysis (27) were fulfilled this method was used to investigate how much variance in itch catastrophizing could be predicted by itch intensity and the mindfulness subscales related to itch catastrophizing in the correlation analyses (“method of best predictors”). In the regression model, the control variables age and sex were entered in the first step of the analysis, itch intensity during the last 2 weeks was entered in the second step and the mindfulness subscales were entered in step three of the analysis.

## Results

### Sample Characteristics

One hundred and sixty three AD patients took part in the study, 8 had to be excluded from the analyses, because they did not fulfill the inclusion criteria. Thus, the final sample size consisted of 155 AD patients (58 men and 97 women). Their mean age (data available from all *n* = 155) was 46.5 ± 12 years. Their mean AD severity (data available from *n* = 135 patients) measured by the PO-SCORAD was 47.9 ± 19.9, which indicates that on average patients suffered from a moderate AD. Twenty-one patients had mild AD, 72 moderate and 42 severe AD. Patients who reported to have a physical comorbidity did not differ from patients without self-reported physical comorbidity regarding itch-catastrophizing (*p* = 0.763) or itch-intensity (*p* = 0.337). For more information regarding the sample characteristics, please see Table 1.

**Table 1:** Description of the sample (*n* = 155 patients with atopic dermatitis).

**Gender**	**Female**	**Male**	**Missing data**
	**97 (62.6%)**	**58 (37.4%)**	**/**
Age (x ± SD)	46.8 ± 11.5	46 ± 13.0	/
Education	No formal qualification: *n* = 1	No formal qualification: *n* = 2	/
	Secondary school qualification: *n* = 70	Secondary school qualification: *n* = 41	
	College or university degree: *n* = 26	College or university degree: *n* = 15	
Self-reported physical comorbidities	Yes: *n* = 48	Yes: *n* = 38	/
CHIME 1 (x ± SD)	4.49 ± 0.89	4.39 ± 0.86	/
CHIME 2 (x ± SD)	4.84 ± 0.96	4.67 ± 0.81	/
CHIME 3 (x ± SD)	3.97 ± 1.00	3.95 ± 0.89	/
CHIME 4 (x ± SD)	3.25 ± 1.07	3.34 ± 0.99	/
CHIME 5 (x ± SD)	3.48 ± 0.98	3.47 ± 0.89	1
CHIME 6 (x ± SD)	3.75 ± 1.00	3.74 ± 0.68	1
CHIME 7 (x ± SD)	3.84 ± 1.01	3.73 ± 0.72	1
CHIME 8 (x ± SD)	3.91 ± 0.89	3.75 ± 0.81	/
Itch Inten (x ± SD)	5.32 ± 2.68	5.89 ± 2.27	2
Itch Cat (x ± SD)	2.34 ± 0.87	2.33 ± 0.95	5
PO-SCORAD (×± SD)	46.69 ± 20.55	49.74 ± 18.96	20

*Itch Cat, Itch Catastrophizing; Itch Inten, Itch Intensity; values for the CHIME-scales can range from 1 to 6 and values for Itch Cat can range from 0 to 4. x, mean; SD, standard deviation*.

*CHIME 1: awareness toward internal experiences*.

*CHIME 2: awareness toward external experiences*.

*CHIME 3: acting with awareness*.

*CHIME 4: accepting and non-judgemental orientation*.

*CHIME 5: non-reactivity*.

*CHIME 6: openness to experiences*.

*CHIME 7: relativity of thoughts*.

*CHIME 8: insightful understanding*.

### Results of the Correlation Analyses

The Pearson correlation analyses revealed that itch catastrophizing and itch intensity during the last 2 weeks were significantly positively related (*r* = 0.409). Moreover, itch catastrophizing was significantly negatively related to the mindfulness subscales acting with awareness (*r* = −0.373), accepting and non-judgemental orientation (*r* = −0.198) and non-reactivity (*r* = −0.260). For the full correlation matrix, please see Table 2. Spearman-Rank-correlations also revealed significant correlations between itch-catastrophizing and the mindfulness subscales “acting with awareness,” “accepting and non-judgemental orientation,” and ‘‘non-reactivity” (*p* < 0.05).

**Table 2:** Results of the *correlation* analyses.

		**0: Itch Cat**	**1**	**2**	**3**	**4**	**5**	**6**	**7**	**8**	**9**	**10**
1	Itch Inten	0.409**	1									
2	CHIME 1	0.039	−0.071	1								
3	CHIME 2	−0.088	−0.044	0.635***	1							
4	CHIME 3	−0.373***	−0.130	0.190*	0.250**	1						
5	CHIME 4	−0.198*	−0.129	0.243**	0.333***	0.352***	1					
6	CHIME 5	−0.260**	−0.053	0.383***	0.503***	0.375***	0.577***	1				
7	CHIME 6	0.084	−0.085	−0.003	0.054	−0.264***	−0.214**	−0.029	1			
8	CHIME 7	0.006	−0.087	0.425***	0.359***	0.000	0.138	−258**	0.189*	1		
9	CHIME 8	−0.028	−0.074	0.401***	0.445***	0.014	0.261***	0.441***	0.163*	0.608***	1	
10	Age	−0.157	−0.166*	−0.034	0.119	0.189*	0.041	0.154	−0.109	−0.063	−0.006	1
11	Sex	0.004	−0.109	0.059	0.089	0.012	−0.040	0.005	0.006	0.062	0.089	0.031

*Shown are correlations between itch catastrophizing, average itch intensity during the last 2 weeks, the eight mindfulness scales measured by CHIME and sex and age. Bold numbers indicate significant correlations*.

*** *Indicate that the correlation is significant on the 0.001 niveau*,

** indicate that the correlation is significant on the 0.01 niveau, *indicates that the correlation is significant on the 0.05 niveau.

*The number of patients included in the analyses varies between n = 149 and 155*.

*Itch Cat, Itch Catastrophizing; Itch Inten, Itch Intensity*.

*CHIME 1: awareness toward internal experiences*.

*CHIME 2: awareness toward external experiences*.

*CHIME 3: acting with awareness*.

*CHIME 4: accepting and non-judgmental orientation*.

*CHIME 5: non-reactivity*.

*CHIME 6: openness to experiences*.

*CHIME 7: relativity of thoughts*.

*CHIME 8: insightful understanding*.

### Results of the Regression Analyses

**Figure 1:**
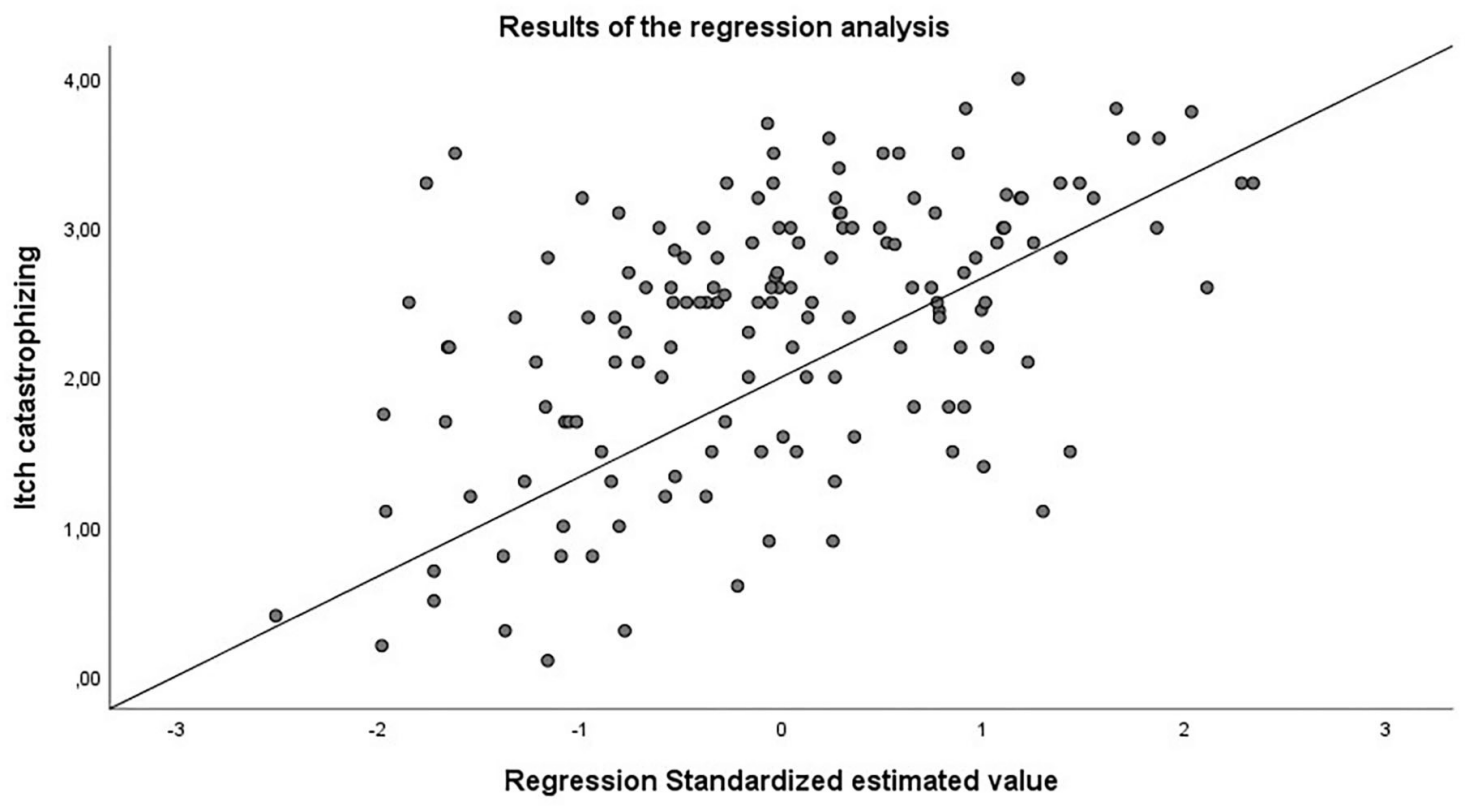
Results of the regression analysis. 27.4% of itch catastrophizing could be explained by itch intensity during the last 2 weeks and the mindfulness scale “acting with awareness”.

The regression analysis showed that 27.4 % of the variance of itch catastrophizing could be predicted by itch intensity and the mindfulness-scale “acting with awareness” (*R*^2^ = 0.284; corrected *R*^2^ = 0.274; *F* = 28.728; *p* < 0.001). Hereby, 17.1% of the variance were explained by itch-intensity and 10.3% by the mindfulness-scale “acting with awareness” (also see Figure 1).

## Discussion

The current study is the first to examine the relationship between mindfulness and itch and mindfulness and itch-catastrophizing in patients with AD. There was no significant relationship between itch intensity and mindfulness; however, there was a significant negative relationship between the mindfulness scale “acting with awareness” and itch catastrophizing.

This finding is in line with the results of previous studies in which mindfulness and especially the mindfulness facets “acting with awareness” and non-reactivity were related to pain catastrophizing and other types of psychological distress (12, 19). This is an important finding as catastrophic thoughts and physiological reactivity are important maintaining factors to itch (6). Furthermore, studies using itch induction suggest that a focus on bodily sensations and catastrophizing of upcoming itch stimuli lead to greater itch intensity or more profound scratching behavior (28, 29).

Our findings suggest that acting with awareness, essentially being more aware of bodily sensations and not reacting to them automatically, may reduce the likelihood of automatic scratching in response to itch. This in turn could then lead to less negative thinking about the skin as skin lesions would not occur that often anymore. In this context, it has already been shown that higher levels of present moment awareness can reduce negative thinking, a characteristic of depression and anxiety (30).

Intervention studies have shown promising results for mindfulness interventions in patients living with long term health conditions and chronic pain (31, 32). In dermatology, mindfulness interventions have been used in patients living with psoriasis with promising results (13, 14, 33). Audio-guided meditations used as an adjunct to light treatment increased the rates of skin clearing (13), and a structured 8-week mindfulness intervention, Mindfulness-based cognitive group therapy, was found to improve dermatology quality of life and self-reported psoriasis severity (14). In atopic dermatitis first results of positive effects in a small patient therapy group were demonstrated (34). However, D'Alton et al. (33) conducted a randomized controlled trial of mindfulness-based interventions in psoriasis and found that whilst participants reported perceiving the interventions as beneficial, there was no significant difference found on distress, symptom burden, or quality of life at completion, or at 6 or 12-month follow-up. Clearly, whilst mindfulness interventions show promise, and our study further supports the theoretical rationale for using it as an intervention where itch is a factor, further examination of mindfulness interventions in a range of skin conditions is required.

### Limitations

There are limitations to the current study. First, this study is a cross-sectional study and therefore conclusions on causation cannot be drawn. Randomized, controlled trials in which “acting with awareness” as one important facet of mindfulness is manipulated by participation in a mindfulness based intervention could provide answers on causal relationships between mindfulness and itch/itch catastrophizing. Further, as participants were recruited at a rehabilitation clinic and were experiencing moderate or severe AD in most cases, the generalisability of our findings to community samples needs to be acknowledged, and be investigated in future studies. In addition, given that AD is known to be associated with anxiety and depression future studies might investigate the role that both “acting with awareness” and catastrophizing play in significant psychological distress associated with AD.

## Conclusion

The current study findings indicate that higher levels of a specific facet of mindfulness termed “acting with awareness” are related to lower levels of itch catastrophizing. This finding is important as it suggests that psychological interventions should seek to increase acting with awareness in patients with AD as a way of improving patient outcomes associated with problematic itch.

## Data Availability Statement

The raw data supporting the conclusions of this article will be made available by the authors upon request.

## Ethics Statement

The studies involving human participants were reviewed and approved by Ethics Committee of the Department of Medicine at Justus-Liebig-University. The patients/participants provided their written informed consent to participate in this study.

## Author Contributions

KL collected the data in this project, contributed to the statistical analyses, and presentation of the results. KM contributed to the first draft of the paper and analyses of the data. AT contributed to study design, writing the theoretical background, and discussion section of the article. UG contributed to the study design. CZ contributed to data collection. JK contributed to the study design and the analyses of the data. CS contributed to the study design, statistical analyses, presentation of the study results and writing the manuscript. All authors critically reviewed the manuscript.

## Conflict of Interest

The authors declare that the research was conducted in the absence of any commercial or financial relationships that could be construed as a potential conflict of interest.
